# MicroRNA 157-targeted *SPL* genes regulate floral organ size and ovule production in cotton

**DOI:** 10.1186/s12870-016-0969-z

**Published:** 2017-01-10

**Authors:** Nian Liu, Lili Tu, Lichen Wang, Haiyan Hu, Jiao Xu, Xianlong Zhang

**Affiliations:** National Key Laboratory of Crop Genetic Improvement, Huazhong Agricultural University, Wuhan, Hubei 430070 China

**Keywords:** Floral organ size, *Gossypium hirsutum*, MADS-box transcription factor, miR157, *SPL* gene, Ovule production

## Abstract

**Background:**

microRNAs (miRNAs) have been involved in regulation of diverse spectrum of plant development processes in many species. In cotton, few miRNAs have been well characterised in floral organ development. Floral organ, which should be finely tuned, is a crucial factor affecting the yield of cotton. Therefore, it is well worth revealing the function of miRNAs in regulation of floral organ development. Here, we report the role of miRNA156/157 in regulation of floral organ size in cotton.

**Results:**

Over-expression of the GhmiRNA157 precursor in cotton (*Gossypium hirsutum*) resulted in smaller floral organs, fewer ovules and decreased seed production due to suppression of cell proliferation and cell elongation. Five *SQUAMOSA promoter-binding protein-like* (*SPL*) genes were identified as targets of GhmiRNA157 using a RNA ligase-mediated rapid amplification of cDNA end approach, and the expression level of miR157-targeted *GhSPL*s decreased in the miR157 over-expression lines, indicating the presence of the miR157/*SPL* axis in cotton. Two MADS-box genes, orthologs of *AtAGL6* and *SITDR8*, which are associated with floral organ development and reproductive production, were repressed in the miR157 over-expression lines. In addition, auxin-inducible genes were also down-regulated, and auxin signal visualized by a DR5::*GUS* reporter was attenuated in the miR157 over-expression lines.

**Conclusions:**

Our results indicate that the miR157/*SPL* axis controls floral organ growth and ovule production by regulating MADS-box genes and auxin signal transduction. The work further elucidates the mechanism of floral organ development and provides helpful molecular basis for improvement of cotton yield.

**Electronic supplementary material:**

The online version of this article (doi:10.1186/s12870-016-0969-z) contains supplementary material, which is available to authorized users.

## Background

Seed number is an important factor in crop yield. Because seeds are derived from fertilized ovules, development of the floral organs, especially the gynoecium, which bears ovules, directly affects the final seed number. Although environmental factors can modulate floral organ development, intrinsic mechanisms control the final size of the floral organs [1].

After the meristematic primordium is established, organ growth can be divided into two phases: cell division followed by cell expansion [2–5]. Many genes, such as the transcription factors *GRFs/GIFs* and *JAGGED*, cytochrome P450 (*KLU*), ubiquitin receptor *DA1* and the E3 ubiquitin ligases *DA2* and *BB*, regulate cell proliferation rate or control the timing of proliferation arrest to maintain normal cell numbers and final organ size [6–10]. Other genes, such as the transcription factor bHLH and mediator complex subunit 8, regulate the cell expansion rate to control the cell area, which affects organ size and shape [11, 12].

Moreover, hormones, such as jasmonic acid, gibberellin, brassinosteroids, and cytokinin, have been reported to be involved in the regulatory network of organ size [3–5, 13, 14]. Auxin plays a very important role in floral organ size and development. Auxin signals directly induce expression of the gene *AUXIN-REGULATED GENE INVOLVED IN ORGAN SIZE* (*ARGOS*), which further upregulates the downstream gene transcription factor *AINTEGUMENTA* (*ANT*) [15]. Over-expression of *ARGOS* or *ANT* can extend the period of cell division, resulting in larger leaves and floral organs with more seeds per silique in *Arabidopsis*. Conversely, mutation of *ARGOS* or *ANT* decreased the final organ size and seed number [15–17]. In addition, *SMALL AUXIN UP RNA* (*SAUR*) was induced by auxin to promote cell elongation and final organ size [18]. Several auxin response factors, which mediate the transcriptional response to auxin, regulate floral organ size and development. *ARF8* was reported to suppress petal cell proliferation, cell elongation and final petal size through interaction with the bHLH transcription factor [19]. *ARF2* could repress *ANT* transcription to limit cell proliferation and organ size [20]. In addition to controlling floral organ size, auxin also regulates normal development of floral organs. In mutants defective in auxin biosynthesis and transport, the gynoecium is a thin and round stalk with diminished or no valve tissues of the ovary, indicating that auxin is necessary for the early establishment of carpel primordium [21]. *MP* (*ARF5*) which, mediates auxin signalling, could control ovule primordial formation by regulating *ANT*, *CUC1* and *CUC2* expression [22].

MADS-box genes are major players in floral organ differentiation and development. Floral homeotic proteins from MIKC-type MADS-box genes form a combinatorial quaternary complex to control differentiation of the distinct floral organs, which was used to explain the principle of the ABC (D)E model [23, 24]. In addition to floral organ identity, MIKC-type MADS-box genes also control floral organ size and shape by regulating cell division and expansion [25]. Through chromatin immunoprecipitation (ChIP), many growth regulatory genes were shown to be targets of MADS-box transcription factors [26–28]. For example, E class MADS-box gene (SEP3) could directly bind *GRF* genes and *JAGGED* gene, which regulate cell division. In addition, MADS-box transcription factors interact with other transcription factors, such as ARF2 and SPL8 [29].

Three MADS-box transcription factor genes, *APETALA1* (*AP1*), *FRUITFULL* (*FUL*), and *SUPPRESSOR OF CONSTANS OVEREXPRESSION 1* (*SOC1*), were directly induced by miR156/157-targeted *SPLs* [30–32]. By promoting *AP1*, *FUL* and *SOC1* expression, miR156/157-targeted *SPLs* could accelerate phase transition. Different miR156/157-targeted *OsSPLs* have been reported to regulate tiller and panicle architecture and grain size in rice [33–36]. MiR156s with highly similar miR157s are collectively referred to as miR156/157 family. Many studies have reported that miR156/157 family could regulate root development, increase tolerance to heat or salt stress, and promote trichome distribution and shoot regenerative capacity [37–40]. Here, we identified another role of miR156/157 family in the regulation of floral organ growth and ovule production through over-expression of a miR157 precursor in cotton. At least five miR156/157-targeted *SPLs* and two MADS-box transcription factors, which are orthologs of *AtAGL6* and *SITDR8*, were down-regulated in the over-expression lines. Meanwhile, auxin signalling was attenuated in the miR157 over-expression lines. We hypothesized that the miR157/*SPL* axis may regulate MADS-box transcription factors and affect auxin signal transduction, finally regulating floral organ growth and ovule production.

## Results

### Over-expression of GhmiR157 in cotton reduced flower size and seed production and altered plant architecture

MiR156/157 family is one of the most conserved miRNA families in the plant kingdom and has many functions in plant development [41, 42]. Through small RNA sequencing, the GhmiR156/157 family was profiled in cotton [43–46].

**Fig. 1:**
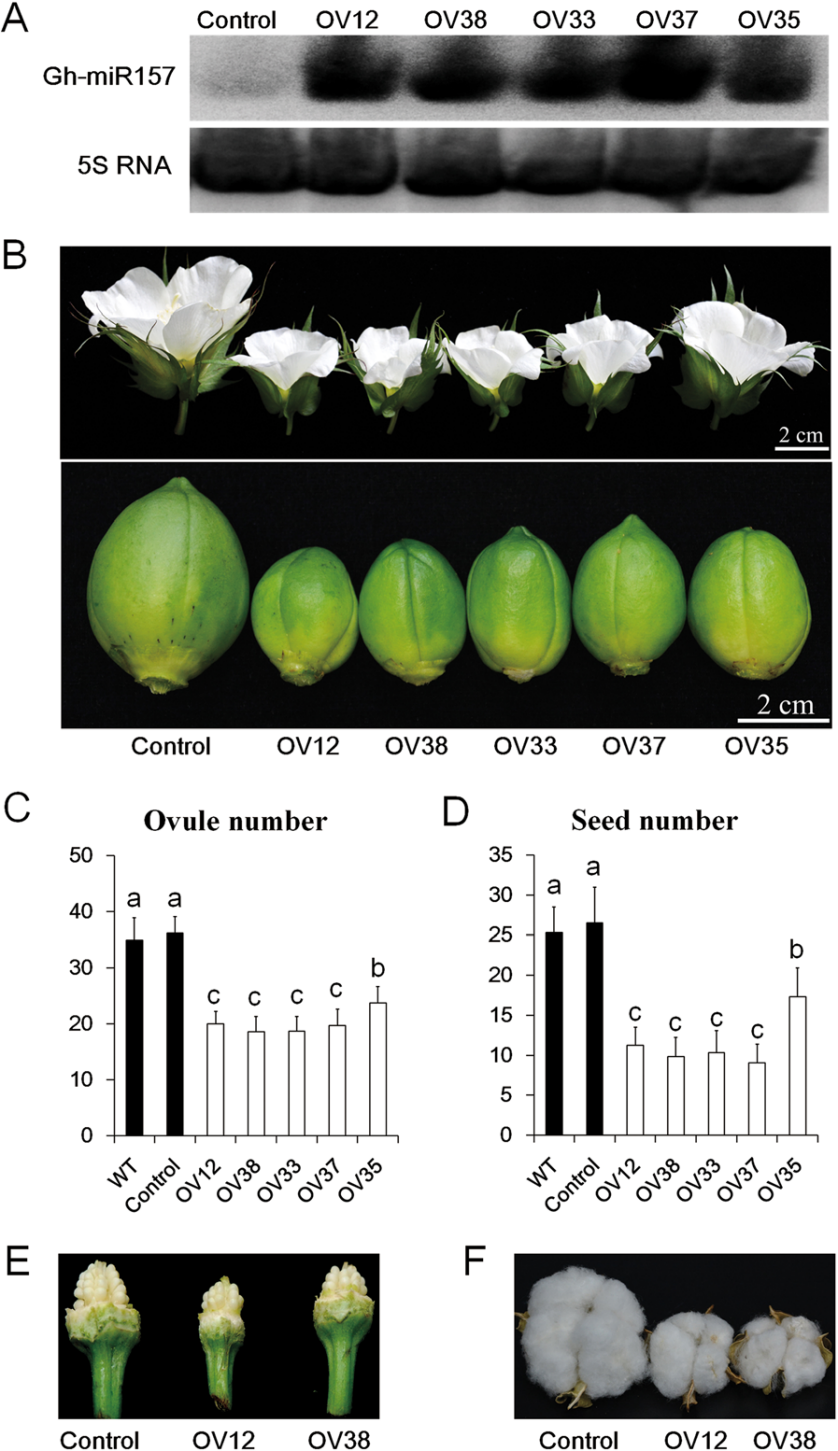
Over-expressing the *GhmiR157* precursor leads to smaller flowers and fewer seeds. **a** Northern blot analysis of mature miR157 expression in floral buds.5S RNA was used as the RNA loading control. **b** Images of opened flowers and 30 DPA bolls. **c** and **d** The number of ovules per ovary and seed numbers per boll. Different letters indicate statistically significant differences at *P* < 0.05 based on analysis of variance (ANOVA) (Tukey’s multiple comparison test). The error bars indicate the standard deviation of at least 20 biological replicates. **e** and **f** Images of ovaries after removing the valves and mature bolls. OV12, 38, 33, 37 and 35, 35S::*GhmiR157* transgenic lines. Control, nontransgenic plant segregated from 35S::*GhmiR157* transgenic lines. WT, wild type (*Gossypium hirsutum* cv. YZ1)

To further analyse the function of GhmiR156/157 family in cotton, we selected a GhmiR157 whose abundance is the highest in GhmiR156/157 family for analysis, and then we cloned a 372 bp genomic sequence containing a miR157 precursor from *Gossypium hirsutum* (Additional file 1) using the predicted reference sequence for *Gossypium raimondii* [46]. To overexpress *GhmiR157*, we used the 35S promoter to drive the miR157 precursor and transferred the construct into YZ1 (*Gossypium hirsutum*). Seven transformants were obtained (Additional file 2), among which five showed dramatically increased mature miR157 expression levels (Fig. 1a). Compared with the nontransgenic plants (Control), the over-expression lines (OV12, 33, 35, 37, 38) showed a stronger vegetative growth vigor and produced more vegetable branches and leaves (Additional file 3). However, the size of the opened flowers and bolls from over-expression lines was notably smaller (Fig. 1b and Additional file 4B, E). In addition, the numbers of ovules per ovary were significantly decreased in the over-expression lines compared with Control, which further led to reduced seeds per boll in over-expression lines (Fig. 1c-f and Additional file 4 D, F). Therefore, we concluded that over-expressing *GhmiR157* altered plant architecture and reduced reproductive capacity sharply.

### Over-expression of GhmiR157 in cotton suppressed floral organ development

**Fig. 2:**
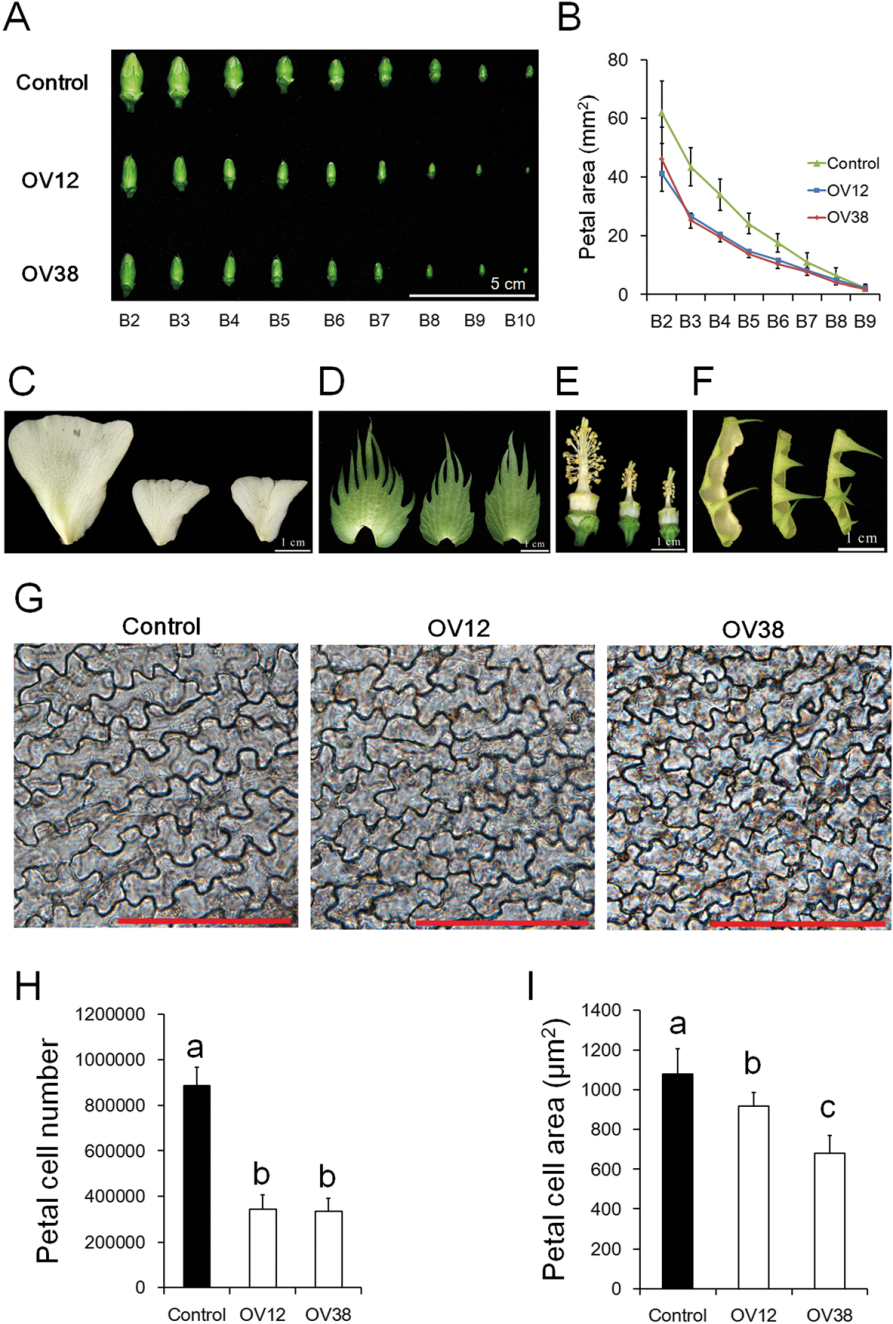
Over-expressing *GhmiR157* suppressed floral organ development. **a** Image of developing floral buds from different fruit branches. **b** Kinematic analysis of the developing petal area. **c**-**f** Images of the petal (**c**), bract (**d**), stamen and gynoecium (**e**) and sepal (**f**) from opened flowers of Control (*left*), OV12 (*middle*) and OV38 (*right*) plants. g Images of epidermal cells at the adaxial side of mature petals. Scale bar, 100 μm. **h** and **i** The number of mature petal cells (**h**) and the mature petal cell area (**i**). B2-B10 represent the position of the developing floral buds from the second to tenth fruit branch. Control, nontransgenic plant segregated from 35S::*GhmiR157* transgenic lines in cotton. OV12 and OV38, 35S::*GhmiR157* transgenic lines in cotton. Different letters indicate statistically significant differences at *P* < 0.05 based on analysis of variance (ANOVA) (Tukey’s multiple comparison test). The error bars indicate the standard deviation of at least eight biological replicates

Because the size of the opened flowers was significantly smaller in over-expression lines, we further compared the size of flower buds at different developmental stages among OV12, OV38 and Control (Fig. 2a). When the first-node flower on the first branch was open, we sampled first-node buds from the second branch (B2) to the tenth branch (B10) in order. Thus, buds from different branches were at different developmental stages. The results showed that at very early emergence (B10), the size of the flower buds had become smaller in OV12 and OV38. Kinematic analysis of petal size also showed that petal development was arrested in OV12 and OV38 after flower bud emergence (Fig. 2b). In addition to petals, the other floral organs, including the bract, sepal, stigma and stamen, were also examined. The areas of the bracts, sepals and petals from opened flowers were smaller in over-expression lines (Fig. 2c, d, f, Table 1 and Additional file 4G). The anther number and the stigma length were also smaller in over-expression lines (Fig. 2e, Table 1 and Additional file 4G). These data illustrated that the development of the four whorls of floral organs was suppressed in the over-expression lines. Because the cell number and cell size are both determinant factors of the final organ size, the epidermal cells at the adaxial side of the mature petals were examined (Fig. 2g-i). The lower cell number and smaller cell size in the over-expression lines demonstrated that cell proliferation and expansion were suppressed in over-expression lines.

**Table 1:** Size of floral organs in Null and miR157 over-expressing lines

	Control	OV12	OV38
Petal area (mm^2^)	965.29 ± 67.83^a^	328.77 ± 87.07^b^	258.49 ± 59.48^b^
Bract area (mm^2^)	553.71 ± 72.36^a^	339.60 ± 110.91^b^	342.29 ± 83.92^b^
Stigma length (mm)	19.26 ± 0.99^a^	10.43 ± 1.26^b^	10.85 ± 1.23^b^
Anther number	66.56 ± 6.68^a^	17.60 ± 3.22^b^	17.64 ± 2.87^b^
Sepal length (mm)	27.80 ± 1.57^a^	20.87 ± 2.42^b^	19.85 ± 1.68^b^
Sepal width (mm)	16.87 ± 2.75^a^	12.69 ± 1.30^b^	11.77 ± 3.11^b^

Control is the nontransgenic plant segregated from the 35S::GhmiR157 transgenic lines in cotton. OV12 and OV38 are 35S::GhmiR157 transgenic lines in cotton. Values are shown as the mean ± standard deviation. In each row, values with different superscipt letters are significantly different based on Tukey’s multiple comparison test (*P* < 0.05)

### Ectopic expression of a GhmiR157 precursor in Arabidopsis also arrested flower development

**Fig. 3:**
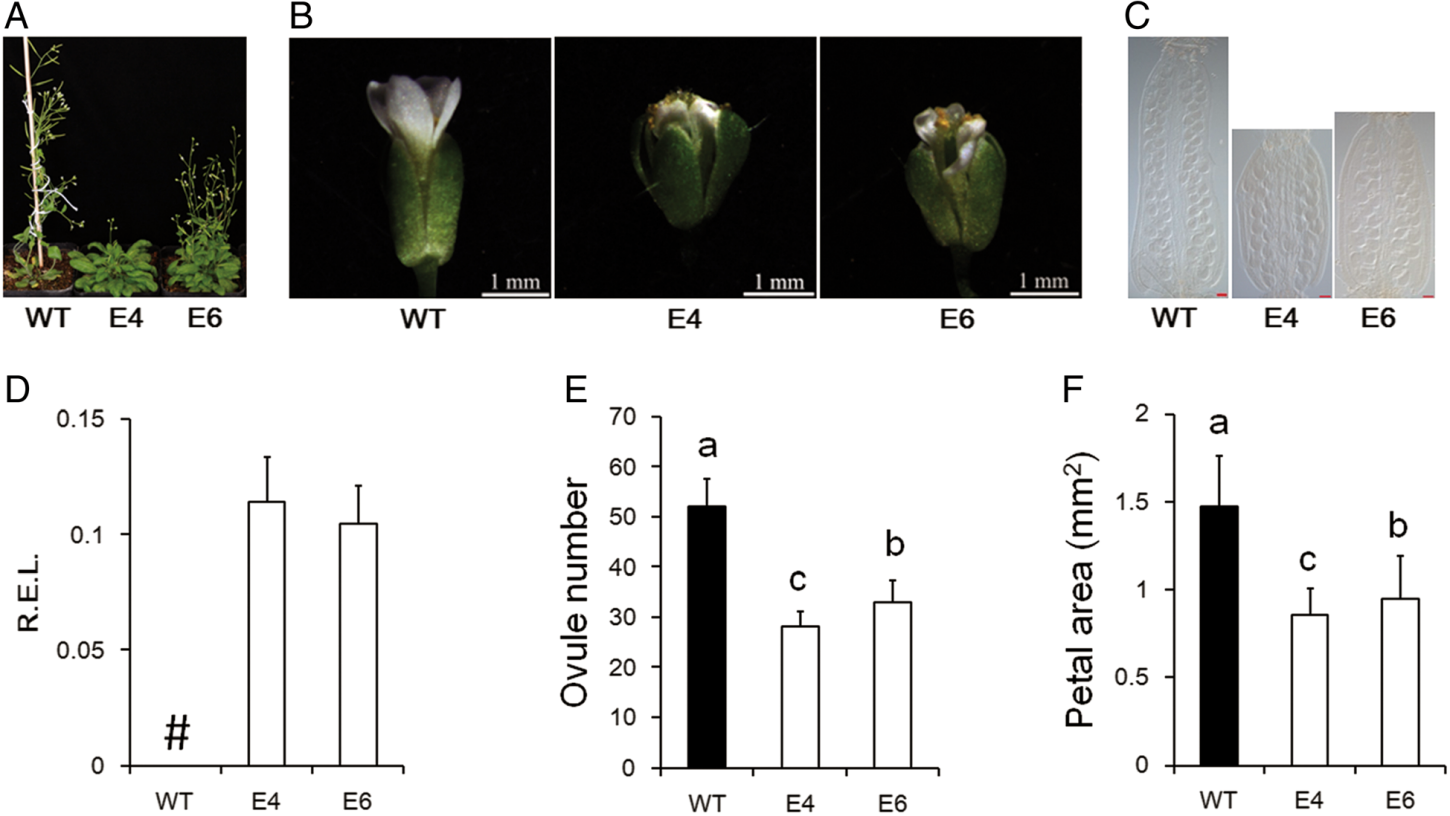
Ectopic expression of the GhmiR157 precursor in *Arabidopsis*. **a** Phenotypes of 35S::*GhmiR157* transgenic lines in *Arabidopsis*. **b** and **c** Phenotypes of flowers and gynoecium at stage 13. Scale bar (**c**), 100 μm. **d** Relative expression of the GhmiR157 precursor in inflorescences. “#”, indicates no expression. R.E.L., the relative expression levels calculated using *AtACT7* (AT5G09810.1) as a control. The error bars indicate the standard deviation of four biological replicates. **e** and **f** The number of ovules per ovary (**e**) and the petal area (**f**). Different letters indicate statistically significant differences at *P* < 0.05 based on analysis of variance (ANOVA) (Tukey’s multiple comparison test). The error bars (**e** and **f**) indicate the standard deviation of at least 15 biological replicates. E4 and E6 indicate 35S::*GhmiR157* transgenic lines in *Arabidopsis*

The miR156/157 family is highly conserved in the plant kingdom [47]. To verify whether the function of miR157 in floral organ development is universal in other species, the GhmiR157 precursor was ectopically expressed in *Arabidopsis*. Two successful ectopic expression lines (E4 and E6) were analysed, and the transgenic lines showed high expression of the miRNA157 precursor (Fig. 3d). Compared to the wild type, E4 and E6 had more rosette leaves (Fig. 3a), and floral organ size, especially petals, was smaller in E4 and E6 (Fig. 3b and f). Meanwhile, the shorter gynoecium contained fewer ovules in E4 and E6 (Fig. 3c and e). Thus, ectopic expression of GhmiR157 in *Arabidopsis* almost reproduced the phenotype in cotton, which indicated that the function of the miR156/157 family in floral organ development is conserved between cotton and *Arabidopsis*.

### Over-expression of GhmiR157 in cotton attenuated female fertility

**Fig. 4:**
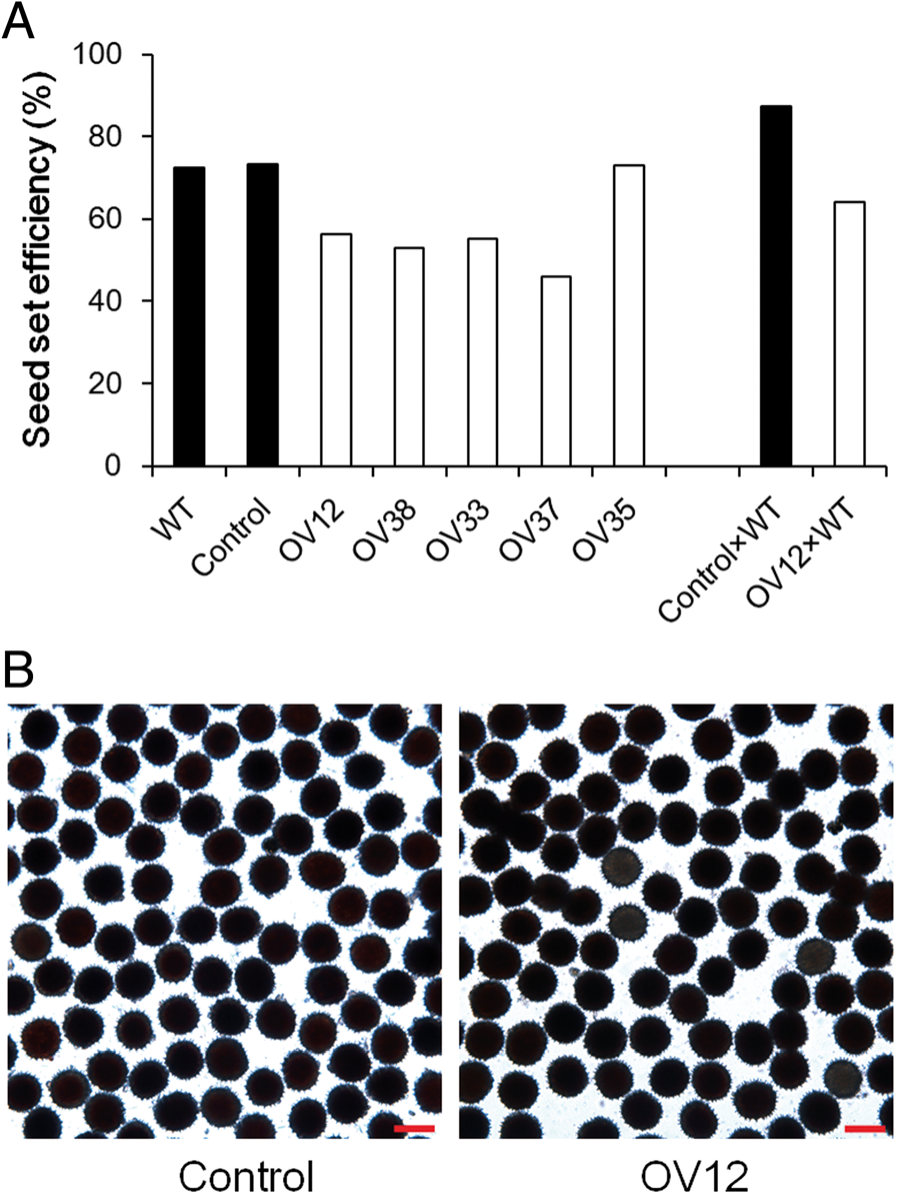
Fertility test of 35S::*GhmiR157* transgenic lines. **a** Calculation of seed set efficiency (100% × seed/ovule). **b** Image of pollen stained with 2,3,5-triphenyltetrazolium chloride. Control, nontransgenic plant segregated from 35S::*GhmiR157* transgenic lines. OV12, 38, 33, 37 and 35, independent 35S::*GhmiR157* transgenic lines. WT, wild type (*Gossypium hirsutum* cv. YZ1). Control × WT and OV12 × WT indicate the plants from Control and OV12 were pollinated with wild type pollens in the greenhouse

Because over-expressing *GhmiR157* arrested floral organ development, we assessed whether gamete fertility was affected. Therefore, we first examined the seed set status among wild type, Control and over-expression lines in the field. The seed set efficiency of the wild type and Control was approximately 70%, but over-expression lines, except OV35, had reduced efficiency, e.g., 46 to 56% (Fig. 4a), indicating that gamete fertility was defective in the over-expression lines. To determine whether the female or male fertility was defective in over-expression lines, we then used wild type pollens to pollinate Control and OV12 plants in the greenhouse. The seed set efficiency was approximately 87% in the Control × WT plants but reduced to 64% in the OV12 × WT plants, demonstrating that female fertility was reduced in the over-expression lines (Fig. 4a). Then 2,3,5-triphenyltetrazolium chloride was used to test pollen vigor, but there was no obvious difference between Control and OV12 (Fig. 4b). Thus, reduced female fertility, not male fertility, resulted in the decrease in seed set efficiency and the reduction of final seed number in over-expression lines.

### RNA-sequencing analysis of developing floral buds

**Fig. 5:**
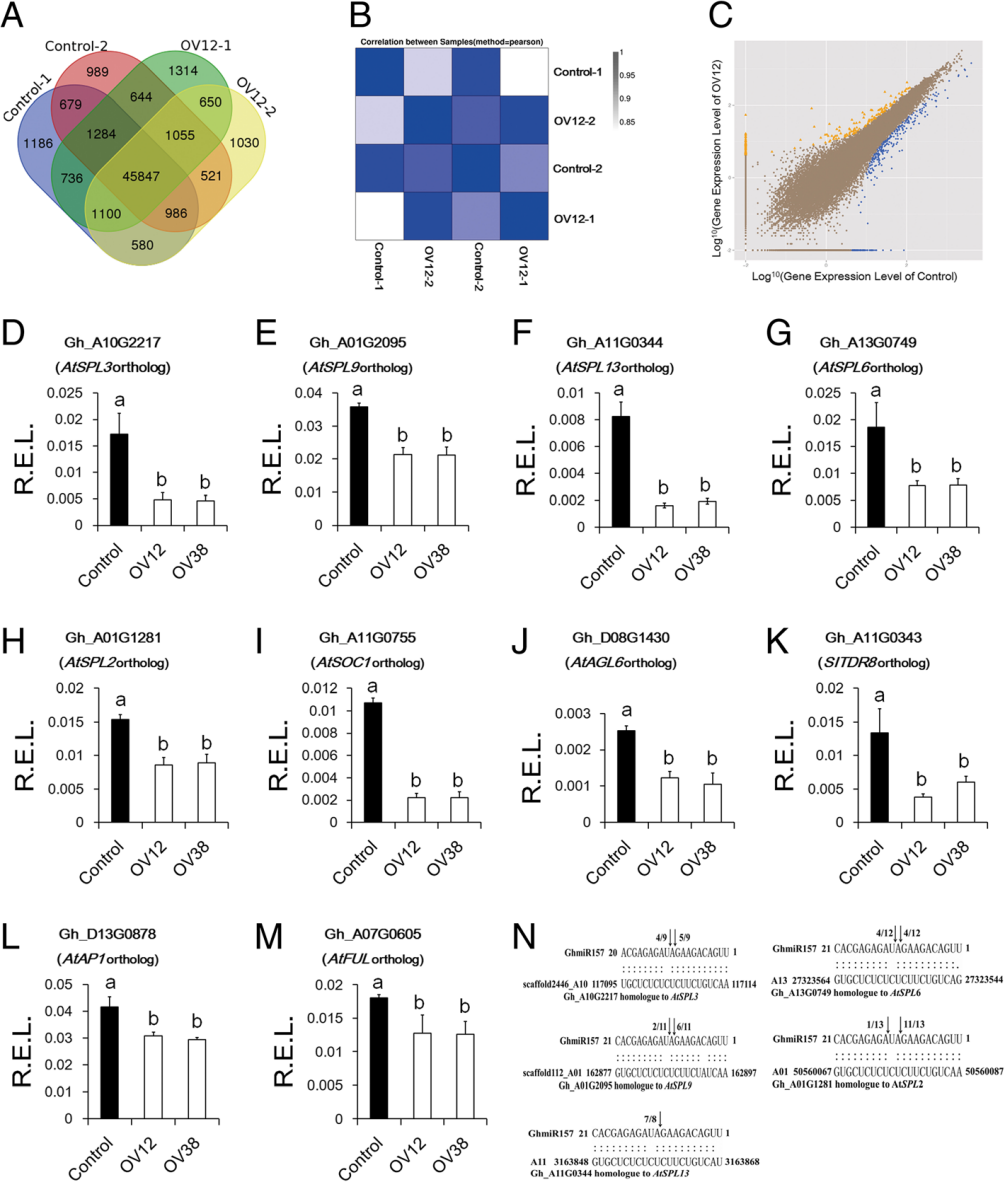
RNA sequencing analysis of differentially expressed genes between Control and 35S::*GhmiR157* transgenic line. **a** Venn diagram of co-expressed genes among samples. **b** Analysis of the correlation between samples. **c** Detection of differentially expressed genes between Control and 35S::*GhmiR157* transgenic line based on the NOISeq method. Yellow plots indicate up-regulated genes in Control, blue plots indicate down-regulated genes in Control, brown plots indicate no changed genes. **d**-**m** Real-time PCR analysis of differentially expressed genes in floral buds. R.E.L., the relative expression levels calculated using *HISTONE3* (AF024716) as a control. The error bars indicate the standard deviation of four biological replicates. Different letters in the plots indicate statistically significant differences at *P* < 0.05 based on analysis of variance (ANOVA) (Tukey’s multiple comparison test). **n** GhmiR157 targets were identified using RLM-RACE. Black arrows indicate the position of the target cleavage sites. The numbers next to the black arrows indicate the cleavage frequency. Control, nontransgenic plant segregated from 35S::*GhmiR157* transgenic lines in cotton. OV12 and OV38, 35S::*GhmiR157* transgenic lines in cotton

To explore how over-expressing *GhmiR157* suppressed floral organ development, RNA-sequencing was performed to identify differentially expressed genes between the over-expression line (OV12) and Control. Because the size of the flower buds was different between Control and OV12 at very early emergence, floral buds (length ≤ 2 mm) were sampled for RNA-sequencing analysis. Approximately 12 million clean reads were generated from two biological repeat libraries of OV12 and Control (Additional file 5). More than 85% of all the clean reads could be mapped to the cotton genome [48], and 52,005 to 52,398 genes were expressed in the libraries from the OV12 and Control plants (Additional file 5). Furthermore, 45,847 genes were expressed in all the libraries, and only 679 and 650 genes were specifically expressed in Control and OV12, respectively (Fig. 5a). The correlations of gene expression level between two biological repeats of OV12 and Control were both more than 0.97, which indicates that the biological repeats are credible (Fig. 5b). To screen differentially expressed genes between Control and OV12, we used the NOISeq package [49]. Genes were filtered based on following criteria: fold change ≥ 2 and divergence probability ≥ 0.8. We identified 539 differentially expressed genes, in which 368 genes were up-regulated in Control, but only 171 genes were down-regulated in control (Additional file 6). Based on another method, the EBSeq package [50], the results also showed that more up-regulated genes were found in Control than in OV12 (Fig. 5c).

In the up-regulated genes of Control, some are transcription factors, such as SPL and MADS-box genes. Interestingly, all the differentially expressed *SPLs* have been predicted as targets of GhmiR157, but the abundance of non-miR157-targeted *SPLs* was not different between Control and OV12 (Additional files 6 and 7). There are 59 members of the SPL family, which could be categorized into eight subgroups based on orthologous genes in *Arabidopsis*, and five of eight subgroups could be predicted as targets of GhmiR157 (Additional files 8 and 7). Five *SPLs*, which were selected from five subgroups, were further verified as GhmiR157 targets using RLM-RACE (Fig. 5n). Real-time PCR was performed to validate the abundance of these five *SPLs*. The results showed that compared to Control, three *SPLs* (Gh_A10G2217, Gh_A11G0344 and Gh_A13G0749) were significantly down-regulated in OV12 and OV38, and the expression level of other *SPLs* (Gh_A01G2095 and Gh_A01G1281) modestly decreased in OV12 and OV38 (Fig. 5d-h). These data demonstrated that GhmiR157-targeted *SPLs* were generally down-regulated in over-expression lines.

MADS-box transcription factor genes, such as *AtSOC1*, *AtAP1* and *AtFUL*, were shown to be directly up-regulated by *SPLs* in *Arabidopsis* [30–32]. Our data showed that three MADS-box transcription factors, Gh_A11G0755, Gh_D08G1430 and Gh_A11G0343, which were orthologs of *AtSOC1*, *AtAGL6* and *SITDR8*, respectively, were significantly down-regulated in OV12 and OV38 compared to Control (Fig. 5i-k). The levels of Gh_D13G0878 and Gh_A07G0605, which are orthologs of *AtAP1* and *AtFUL*, were slightly lower in OV12 and OV38 than Control (Fig. 5l and m). Similarly, *AtAP1*, *AtFUL*, *AtSOC1*, and *AtAGL6* were also down-regulated in 35S::*GhmiR157 Arabidopsis* transgenic lines compared with the wild type (Additional file 9). All the data indicated that MADS-box genes, as candidate downstream genes of GhmiR157-targeted *SPLs*, were down-regulated in over-expression lines.

### Expression pattern of GhmiR157 and miR157-targeted GhSPLs during floral bud development

**Fig. 6:**
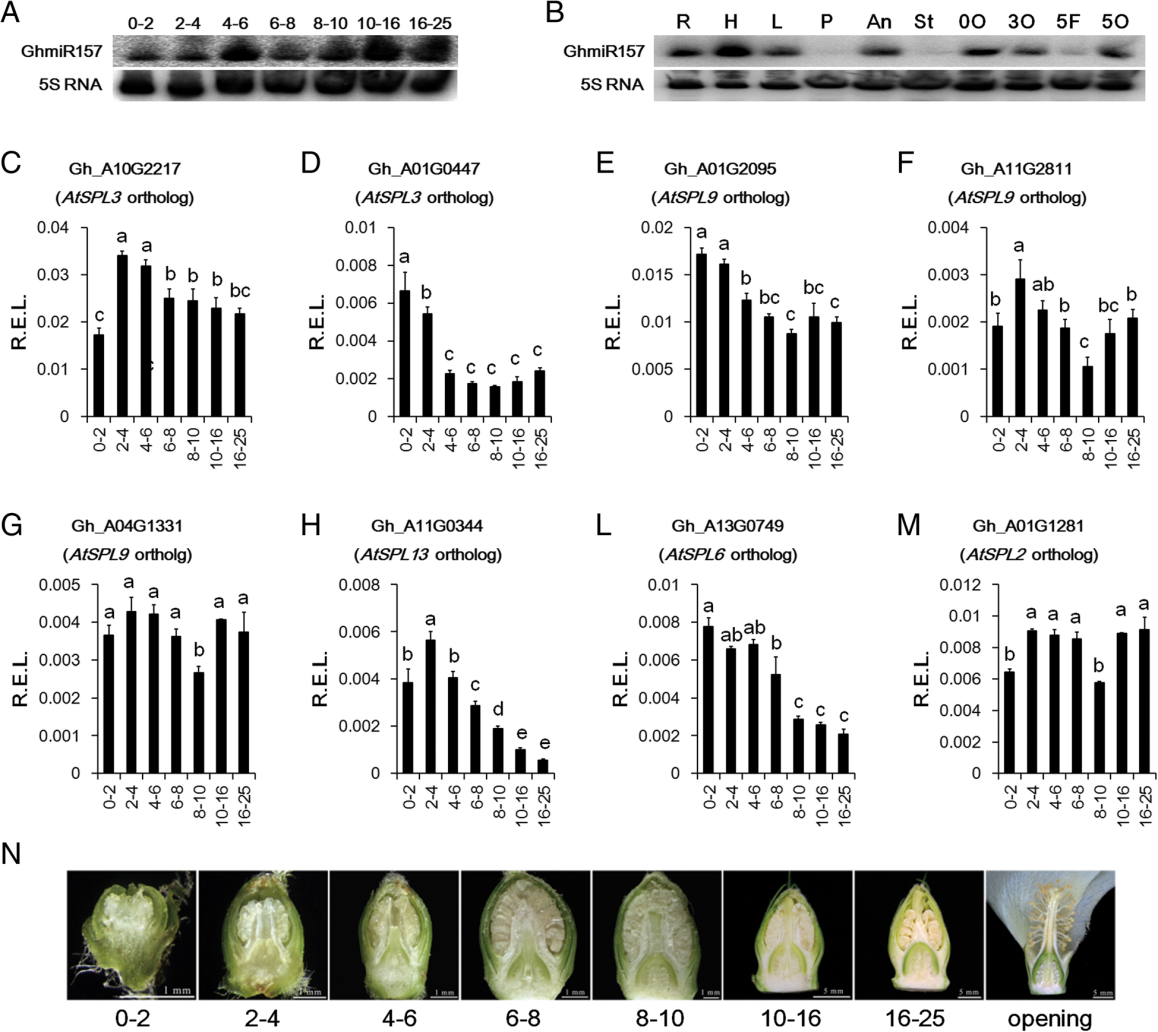
qRT-PCR analysis of GhmiR157 and *GhSPL* expression patterns. **a** Northern blot analysis of mature GhmiR157 expression during floral organ bud developmental stages. **b** Northern blot analysis of mature GhmiR157 expression in different tissues. R, H, L, P, An, St, 0O, 3O, 5 F, 5O represent the root, hypocotyl, leaf, petal, anther, stigma, 0 DPA ovule, 3 DPA ovule, 5 DPA fibre, and 5 DPA ovule, respectively. **c**-**m** Quantitative analysis of *GhSPL* expression in different floral bud developmental stages. R.E.L., the relative expression levels calculated using *HISTONE3* (AF024716) as a control. The error bars indicate the standard deviation of three biological replicates. Different letters in the plots indicate statistically significant differences at *P* < 0.05 based on analysis of variance (ANOVA) (Tukey’s multiple comparison test). **n** Images of longitudinal sections of different length (mm) of floral buds and opened flowers. The numbers 0–2, 2–4, 4–6, 6–8, 8–10, 10–16, 16–25 represent different lengths (mm) of floral buds

Floral organ development in cotton lasts approximately a month from floral bud emergence to flower opening. Based on floral bud length, we identified seven stages of floral bud development before flower opening (Fig. 6n). Ovules appeared at the 4–6 mm floral bud stage. After that, the floral bud, including the differentiated four floral whorls, continued growing to approximately 25 mm in length before the flower opened. GhmiR157 expression was very low at the 0–2 mm and 2–4 mm stages, but it was expressed at much higher levels at the 10–16 mm and 16–25 mm stages. Generally, GhmiR157 abundance increased during floral organ development (Fig. 6a). In opened flowers, GhmiR157 was predominantly expressed in the anther and ovule, indicating that GhmiR157 may play a role in anther and ovule maturation. High levels of GhmiR157 could also be detected in the root, hypocotyl and leaf of seedlings (Fig. 6b).

The expression patterns of eight differentially expressed miR157-targeted *GhSPLs* (Fig. 5 and Additional file 6) were analysed during floral organ development. And statistically significant differences of expression level among floral organ development stages was analysed, based on analysis of variance. Since GhmiR157 could trigger degradation of its targeted mRNAs, the expression pattern of most miR157-targeted *GhSPLs* showed a decreasing trend during floral organ development. Gh_A01G0447, Gh_A01G2095, Gh_A11G0344, and Gh_A13G0749 were obviously down-regulated during the floral bud developmental stages (Fig. 6d, e, h and l). The abundance of Gh_A10G2217 and Gh_A11G2811 also slightly decreased (Fig. 6c and f). However, expression patterns of Gh_A04G1331 and Gh_A01G1281 did not show an obvious trend during floral bud development (Fig. 6g and m). According to negative correlation of expression pattern between GhmiR157 and most miR157-targeted *GhSPLs*, it is reasonable to speculate that GhmiR157 could trigger degradation of its targeted mRNAs to suppress the expression of miR157-targeted *GhSPLs* during floral organ development.

### Auxin signalling was attenuated in the over-expression lines

**Fig. 7:**
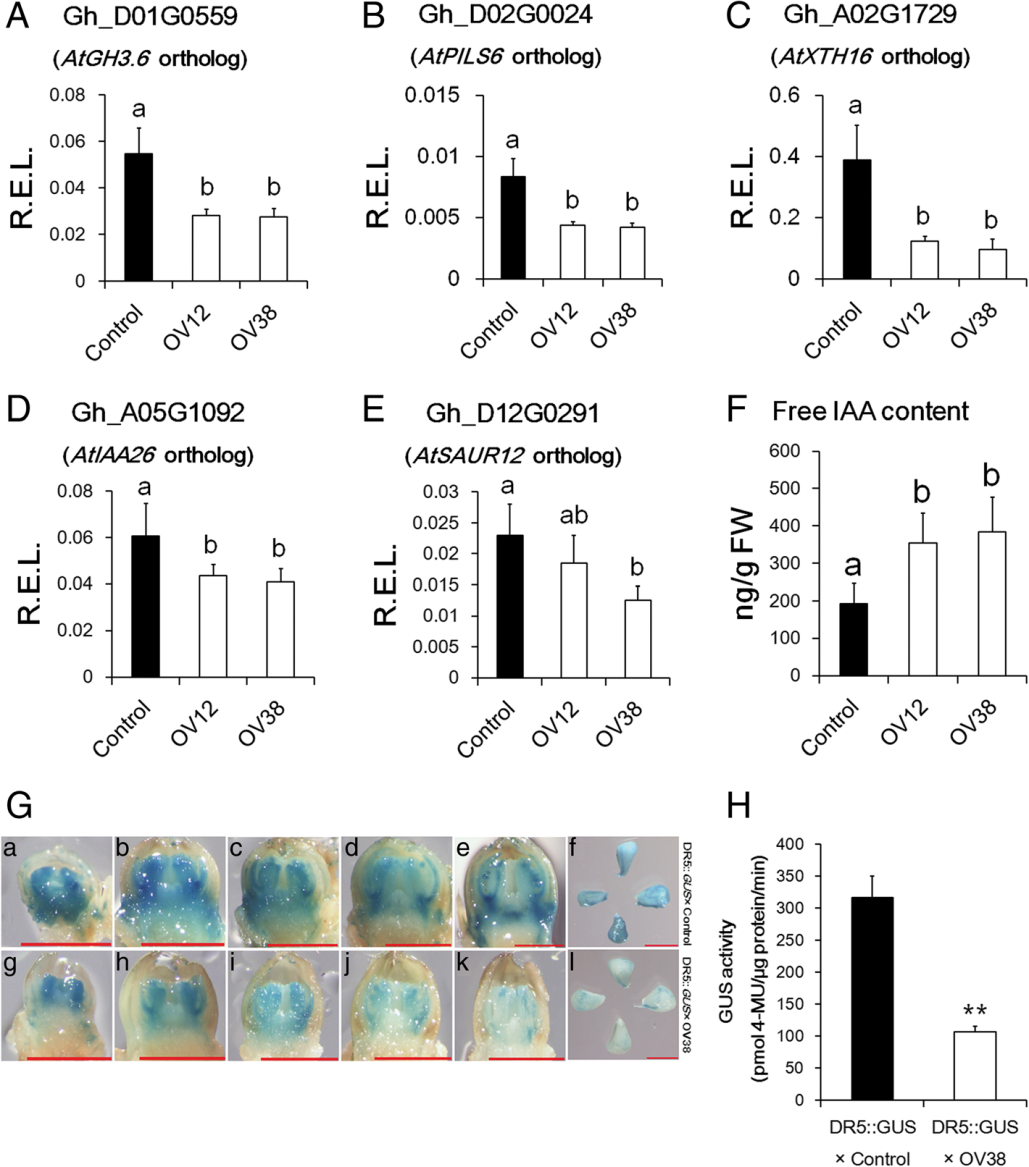
Auxin signalling was lower in over-expression lines than Control. **a**-**e** Real-time PCR analysis of auxin-inducible genes in floral buds. R.E.L., the relative expression levels calculated using *HISTONE3* (AF024716) as a control. The error bars indicate the standard deviation of four biological replicates. Different letters indicate statistically significant differences at *P* < 0.05 based on analysis of variance (ANOVA) (Tukey’s multiple comparison test). **f** Quantitative analysis of IAA content in the floral buds of Control, OV12 and OV38 at −1 DPA. The error bars indicate the standard deviation of at least six biological replicates. Different letters indicate statistically significant differences at *P* < 0.05 based on analysis of variance (ANOVA) (Tukey’s multiple comparison test). **g** GUS staining images of floral buds and ovules. Images of floral buds (length ≤ 2 mm) from DR5::*GUS* × Control (a-e) and DR5::*GUS* × OV38 (g-k) and ovules from DR5::*GUS* × Control (f) and DR5::*GUS* × OV38 (l) at −1 DPA. Scale bar, 1 mm. **h** Quantitative GUS assays of ovules at −1 DPA. Asterisks indicate statistically significant differences at *P* < 0.01 based on Student’s *t* test. Control, nontransgenic plant segregated from 35S::*GhmiR157* transgenic lines in cotton. OV12 and OV38, 35S::*GhmiR157* transgenic lines in cotton. DR5::*GUS* × Control and DR5::*GUS* × OV38 were F1 plants of DR5::*GUS* crossed with Control and OV38, respectively

Auxin plays an important role in flower development and floral organ size [21, 51, 52]. Intriguingly, some auxin-inducible genes, such as IAA-amido synthase, Auxin efflux carrier family protein and xyloglucan endotransglucosylase, were substantially down-regulated in two over-expression lines compared to Control (Fig. 7a-c). Other well-known auxin-inducible genes, IAA and SAUR, also had slightly lower expression in OV38 lines than the Control lines (Fig. 7d and e). To further verify the auxin signal difference between Control and over-expression lines, we used the plants transfected with the DR5::*GUS* vector to cross OV38 and Control plants, respectively. Since the *DR5* promoter consists of seven repeats of an auxin-response element [53], the DR5::*GUS* reporter could be used to monitor the auxin response level. GUS signalling in floral buds and ovules was lower in DR5::*GUS* × OV38 than DR5::*GUS* × Control, indicating that auxin signalling was attenuated in the over-expression lines (Fig. 7g and h). However, transcript abundance of the key auxin biogenesis gene flavin monooxygenase (*YUCCAs*) did not show significant differences between Control and OV38 as determined by the RNA-sequencing results (Additional file 6). Additionally, free IAA content was conversely lower in Control than in OV12 and OV38 (Fig. 7f), which may indicate the existence of feedback regulation because the down-regulated *AtGH3.6* ortholog in over-expression lines may decrease free IAA conjugation with amino acids. Thus, we concluded that auxin signal transduction was attenuated in over-expression lines.

## Discussion

Recently, a lot of miRNAs have been identified through small RNA sequencing in cotton [43–46]. However, few of their function have been verified in cotton. In this study, we found that over-expression of GhmiR157 in cotton could arrest cell proliferation and cell expansion, which repressed floral organ development and reduced the final organ size (Figs. 1 and 2, Table 1 and Additional file 4). Since floral bud growth was arrested at very early emergence, ovule primordium establishment may be repressed in the over-expression line, which resulted in reduced ovule production. All these results demonstrated that GhmiR157 may play an important role in floral organ development, although the mechanism needs to be further elucidated. Ectopic expression of the GhmiR157 precursor in *Arabidopsis* also reduced the petal area, gynoecium length and ovule number, which was similar to the phenotype in cotton. Since short gynoecium and few ovules were also found in *35S:AtMIR156b* transgenic plants in *Arabidopsis* [54], it appears that the regulatory function in floral organ size and growth is conserved between species for miR156/157. Over-expressing the *AtMIR156b* precursor in tomato did not reduce floral organ size but resulted in severe fruit development defects, indicating that the miR156/157 family plays a major role in reproductive organ development among different species [55].

SPL transcription factors have been reported as miR156/157 targets in many species [42], and degradome sequencing also demonstrated that *GhSPLs* are targets of miR156/157 in cotton [43, 56]. Additionally, in all the differentially expressed genes between the over-expression line and Control, only SPL transcription factors were predicted as miR157 targets, and five *GhSPLs* were further verified as miR157 targets using RLM-RACE (Fig. 5n and Additional file 7). The expression patterns of GhmiR157 and several targeted *GhSPLs* were generally negatively correlated during the floral organ developmental stages (Fig. 6). Therefore, it is reasonable to presume that *GhSPLs* as miR157 targets play important roles in floral organ development.

In *Arabidopsis*, AtSPLs could directly promote MADS-box transcription factors, such as *AtSOC1*, *AtAP1*, and *AtFUL*, to control phase transition [30–32]. In our study, the *AtSOC1* ortholog and two other MADS-box transcription factors, the *AtAGL6* ortholog and *SITDR8* ortholog, were significantly down-regulated in the over-expression lines compared to Control (Fig. 5i-k and Additional file 6). The AGL6 clade of MADS-box genes is very similar to the closely related E class MADS-box genes and could serve as a scaffold to interact with other A, B, C and D class MADS-box transcription factors to form combinatorial quaternary complexes [57–59]. Dominant loss of function of the AGL6 clade of MADS-box genes by fusing a conserved suppressing motif to the proteins resulted in much smaller floral organs and partial sterility in *Arabidopsis* [58, 60]. *SITDR8* is another clade of MADS-box genes, which was not found in *Arabidopsis* [61]. Dominant loss of function of *SITDR8* leads to alteration of ovary shape and seedless fruits [62]. MADS-box transcription factors play a crucial role in floral organ differentiation and development, and over-expressing miR157 in cotton resulted in smaller floral organs, fewer ovules and seeds, and attenuated female fertility, which partially reproduced the phenotype of mutants of these two genes in *Arabidopsis* and tomato. Therefore, it is likely that the miR157/SPL axis regulated orthologs of *AtAGL6* and *SITDR8* in cotton to control normal floral organ growth. However, direct regulation of these two clades of MADS-box genes in cotton by miR157-targeted *GhSPLs* should be verified in the future.

In addition to MADS-box transcription factors, several auxin-inducible genes were down-regulated in over-expression lines compared with Control (Fig. 7a–e). Moreover, auxin signalling, which could be monitored by the DR5::*GUS* reporter, was also lower in the over-expression lines than Control (Fig. 7g and h). However, free IAA content was not lower (Fig. 7f). Therefore, decreased auxin signalling in the over-expression lines was not due to IAA content but defects in signal transduction. The mechanism of how the miR157/SPL axis functions in auxin signal transduction is not clear. Interestingly, it was reported that the E class MADS-box transcription factor SEPALLATA3 could bind several ARF recognition motifs, for example, the *AtGH3.3* promoter region [26]. Another study reported that MADS-box transcription factors could interact with ARF2 [29]. Given that AGL6 is functionally similar to SEPALLATA3, and the ortholog of *AtAGL6* is down-regulated in over-expression lines, it is reasonable to hypothesize that MADS-box transcription factors, which may be regulated by miR157-targeted SPLs, possibly serve as ARF-like or ARF partner-like molecules to transduce auxin signalling to regulate normal organ differentiation and growth.

**Fig. 8:**
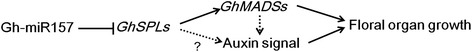
A model for the potential miR157/SPLs axis in the regulatory network of floral organ development. The T shapes represent negative regulation. The dashed lines indicate hypothetical positive regulation

Finally, we propose a possible regulatory network of floral organ growth as described in Fig. 8. At floral bud emergence, there are low levels of GhmiR157 and little degradation of its targets, *GhSPLs* mRNAs. Highly expressed miR157-targeted *GhSPLs* may activate transcription of MADS-box genes, such as orthologs of *AtAGL6* and *SITDR8*. These MADS-box transcription factors, or some unknown factors, may bind auxin response motifs of downstream gene promoters to transduce auxin signalling. Activated auxin signalling and MADS-box genes may further regulate downstream genes to establish normal organ primordium (such as ovules) and promote cell proliferation and cell expansion. In the late floral organ development stage, a high abundance of GhmiR157 decreases *GhSPL* expression to reduce the growth rate and accelerate floral organ (such as anther and ovule) maturation for fertilization.

## Conclusions

Floral organs are crucial factors affecting the harvest of many crops and are complex reproductive organs that are regulated by many transcription factors and hormones [51]. Here, we found that the miR157/SPL axis could affect floral growth and size formation through regulating MADS-box genes and auxin signal transduction. The work further illuminates molecular basis of floral organ development, which is helpful for improvement of cotton yield. Future studies of the crosstalk between the miR157/SPL axis and other factors in the regulation of floral organ development should be performed.

## Methods

### Plant materials and RNA isolation

*Gossypium hirsutum* cv. YZ1 was used as the wild type and transgenic receptor. All transgenic and non-transgenic cotton plants were grown in the experiment field and greenhouse at Huazhong Agricultural University in Wuhan using standard farming management practices in accordance with relevant national approvals for biotechnology research. The floral buds with lengths ≤ 2 mm and flower buds at minus one day post-anthesis (−1 DPA) were harvested, immediately immersed in liquid nitrogen and stored at −80 °C. Total RNA was extracted using a thiocyanate method [63].

*Arabidopsis thaliana* ecotype Columbia was used as the wild type and transgenic receptor. Plants were grown in a greenhouse at 20 °C-22 °C under long-day conditions (16 h light/8 h dark). Inflorescences were harvested, immersed in liquid nitrogen and stored at −80 °C. Total RNA was extracted using TRIzol® reagent according to the protocol (Thermo Fisher Scientific).

### Plasmid construction and genetic transformation

A 372 bp genomic sequence containing a miR157 precursor from *Gossypium hirsutum* (Additional file 1) was cloned and ligated into the pGWB402 vector to overexpress the miR157 precursor [64]. A DR5 promoter fragment was ligated into the pGWB433 vector to construct the DR5::*GUS* vector [64]. The oligonucleotides for generating the plasmids described above are listed in Additional file 10.

*Agrobacterium tumefaciens* (GV3101) carrying the vector was used to transform hypocotyls of *Gossypium hirsutum* cv. YZ1. The infected hypocotyls were treated as described previously [65]. *Agrobacterium tumefaciens* (GV3101)-mediated transformation of *Arabidopsis thaliana* ecotype Columbia plants was performed by the floral dip method [66].

### Southern blotting, northern blotting and qRT-PCR analysis

Southern blotting was performed as follows: genomic DNA isolation, enzyme digestion, electrophoresis and hybridization. The detailed methods were described previously [67]. The PCR-generated *NPTII* fragment was used as the probe. The relevant primers are listed in Additional file 10.

Northern blotting of miRNA was performed according to a previous report [43]. First, 20 μg total RNA was electrophoresed in a 15% denaturing polyacrylamide gel containing 8 M of urea and transferred to an Immobilon-Ny + membrane (Merck Millipore). Then, the probes were labelled with γ32P-ATP using T4 polynucleotide kinase (New England BioLabs). After hybridization and membrane wash, the blot was exposed to a phosphor Imager screen, and the signal was detected in Cyclone Plus Phosphor Imager (PerkinElmer).

To quantify mRNA expression, 3 μg total RNA was reverse-transcribed to cDNA using SuperScript II reverse transcriptase (Invitrogen). qRT-PCR was performed using a 7500 real-time system (Applied Biosystems) with Sso-Fast EvaGreen Supermix With Low ROX (Bio-Rad). The relative expression levels (R.E.L.) were calculated using the 2^-ΔCT^ method. *HISTONE3* (AF024716) and *AtACT7* (AT5G09810.1) were used as endogenous reference genes in cotton and *Arabidopsis* respectively.

### Morphological and cellular analysis

For kinematic analysis of cotton petal development, petals were manually dissected from the first-node floral bud on the second branches back to the first-node floral bud on the ninth branches when the first-node flower on the first branches opened.

For measurements of petal, sepal and bract area, the organs were flattened and scanned to produce a digital image. ImageJ software (https://imagej.nih.gov/ij/download.html) was used to calculate the organ areas. The cotton ovule, seed and anther number and stigma length were calculated manually. For measurement of ovule number in *Arabidopsis*, the gynoecium was washed in 70% ethanol twice and cleared in chloral hydrate:dH_2_O:glycerine (8:3:1). Ovules were observed using differential interference contrast microscopy (ZEISS).

Petal cell size was measured on the adaxial side at the top of petal. Average cell size was calculated from the number of cells per unit area of images from microscopy (ZEISS). Petal cell number was calculated according to cell size and petal area.

### Bioinformatic analysis of sequencing data

Floral buds (length ≤ 2 mm) were sampled from Control and miRNA157-over-expressing OV12 plants growing in a greenhouse. RNA libraries were generated and sequenced via Illumina HiSeq^TM^ 2000 at the Beijing Genomics Institute (BGI) in Shenzhen. After sequencing, the raw reads were filtered into clean reads and then mapped to the reference genome of *Gossypium hirsutum* [48] using *Bowtie* [68]. Gene expression levels were quantified using the software package RNASeq by Expectation Maximization [69]. Differentially expressed genes between the Control and OV12 lines with two biological repeats were screened using the NOISeq package [49].

miR157 target prediction was performed on a website tool using the default criterion [70].

### RNA ligase-mediated rapid amplification of cDNA end (RLM-RACE)

RLM-RACE was performed with a GeneRacer kit (Invitrogen) to map the cleavage sites of target transcripts. Total RNA (5 μg) from floral buds (length ≤ 2 mm) was ligated to RNA adapters without calf intestine alkaline phosphatase. The cDNAs were transcribed using the GeneRacer Oligo dT primer. The PCR was performed with 5ˈ adaptor primers and 3ˈ gene-specific primers according to the manufacturer’s instructions. RACE products were cloned, and approximately 10 inserts were sequenced and analysed.

### Histochemical analysis and quantification of GUS activity

Floral buds (length ≤ 2 mm) and ovules (−1 DPA) were incubated in the GUS staining buffer at 37 °C for 4 h and then washed in 75% ethanol one or more times. Stained samples were photographed using a stereomicroscope (Leica Microsystems). The staining buffer contained 0.9 g L^−1^ 5-bromo-4-chloro-3-indolylglucuronide, 50 mM sodium phosphate buffer (pH 7.0), 20% (v/v) methanol and 100 mg L^−1^ chloromycetin.

For quantification of GUS activity, total protein from the samples was extracted using GUS extraction buffer containing 50 mM potassium phosphate buffer at pH 7.0, 10 mM ethylenediaminetetraacetic acid, 0.1% sodium laurylsarcosine, 0.1% Triton X-100 and 10 mM β-mercaptoethanol. The homogenate was centrifuged, and the supernatant was collected to measure GUS activity as described previously [71].

### Quantification of endogenous IAA

Floral buds (100 mg fresh weight) at −1 DPA were homogenized in 1 mL of 80% (vol/vol) methanol containing 10 ng/mL ^2^H_5_-IAA (OIChemlm Ltd, CAS: 76937-78-5) as the internal standard and then shaken at 4 °C overnight. The supernatant was evaporated and redissolved in 10% (vol/vol) methanol and subsequently filtered through a 0.22 μm nylon membrane. The quantification of endogenous IAA was performed according to a previous report [72].

## Additional files

Additional file 1: GhmiR157 precursor from *Gossypium hirsutum*. (**A**) Genomic sequence containing GhmiR157 precursor. The underline indicates mature miR157 sequence. (**B**) The secondary structure of GhmiR157 precursor. The red line indicates mature miR157 sequence. (DOCX 2223 kb)
Additional file 2: Positive test for 35S::*GhmiR157* transformants. (**A**) Southern blot of 35S::*GhmiR157* transformants. (**B**) PCR analysis of 35S::*GhmiR157* transformants. Control, nontransgenic plant segregated from 35S::*GhmiR157* transgenic lines in cotton. The numbers 11, 12, 33, 35, 37, 38, 40 represent different 35S::*GhmiR157* transgenic lines in cotton. (DOCX 287 kb)
Additional file 3: Over-expressing *GhmiR157* precursor leads to more vegetative branches. (**A**-**E**) The pictures of plants at squaring stage in trial field from Control (**A**), OV12 (**B**), OV38 (**C**), OV33 (**D**), OV35 (**E**). (**F** and **G**) Plants at boll opening stage in field from Control (**F**) and OV12 (**G**). (**H**) Measurement of vegetative branches at squaring stage in green house. Control, nontransgenic plant segregated from 35S::*GhmiR157* transgenic lines. OV12, 38, 33, 37 and 35, independent 35S::*GhmiR157* transgenic lines. Different letters indicate statistically significant differences at *P* < 0.05 based on analysis of variance (ANOVA) (Tukey’s multiple comparison test). (DOCX 748 kb)
Additional file 4: Over-expressing *GhmiR157* precursor suppressed reproductive organs development. (A) qRT-PCR of mature miR157 expression in floral buds. R.E.L., the relative expression levels calculated using *HISTONE3* (AF024716) as a control. The error bars indicate the standard deviation of three biological replicates. Different letters indicate statistically significant differences at *P* < 0.05 based on analysis of variance (ANOVA) (Tukey’s multiple comparison test). (B-F) Images of flowers (B), stamens and stigmas (C), ovaries after removing the valves (D), 30 DPA bolls (E) and mature bolls (F). (G) The size of floral organs in WT and over-expressing *GhmiR157* lines. Values are shown as the mean ± standard deviation. In each column, values with different letters are significantly different based on Tukey’s multiple comparison test (*P* < 0.05). OV12, 38, 33 and 35, independent 35S::*GhmiR157* transgenic lines. WT, wild type (*Gossypium hirsutum* cv. YZ1). Control, nontransgenic plant segregated from 35S::*GhmiR157* transgenic lines. (DOCX 400 kb)
Additional file 5: Summary statistics of sequencing and mapping. (XLSX 10.1 kb)
Additional file 6: The abundance and annotation of genes in Control and OV12. (XLSX 8040 kb)
Additional file 7: Predicted targets of miR157. (XLSX 11.8 kb)
Additional file 8: Unrooted phylogram of all *SPL* genes in *Gossypium hirsutum* and *Arabidopsis*. Unrooted phylogram was performed by MEGA6 software based on the neighbor-joining algorithm. The red lines indicate the subfamily members are candidates of miR157 targets predicted through psRNATarget website tool [69]. (DOCX 48.2 kb)
Additional file 9: Quantification analysis of MADS-box genes in ectopic-expressing GhmiR157 precursor lines and wild type. (A-D) qRT-PCR of MADS-box genes in inflorescence. E4 and E6 indicate 35S::*GhmiR157* transgenic lines in *Arabidopsis*. R.E.L., the relative expression levels calculated using *AtACT7* (AT5G09810.1) as a control. The error bars indicate the standard deviation of four biological replicates. (DOCX 88.3 kb)
Additional file 10: Primer applied in the study. (XLSX 12.7 kb)

## Abbreviations

*AP1*: *APETALA1*
*FUL*: *FRUITFULL*
*SAUR*: *SMALL AUXIN UP RNA*
*SOC1*: *SUPPRESSOR OF CONSTANS OVEREXPRESSION 1*
*SPL*: *SQUAMOSA Promoter- Binding Protein- Like*
