# Supplementary material for: MicroRNA 157-targeted SPL genes regulate floral organ size and ovule production in cotton
Source: BMC Plant Biol. 2017 Jan 10;17:7. doi: 10.1186/s12870-016-0969-z (PMC5223427; doi:10.1186/s12870-016-0969-z)
Supplement: Additional file 1: — GhmiR157 precursor from Gossypium hirsutum. (A) Genomic sequence containing GhmiR157 precursor. The underline indicates mature miR157 sequence. (B) The secondary structure of GhmiR157 precursor. The red line indicates mature miR157 sequence. (DOCX 2223 kb) [file 12870_2016_969_MOESM1_ESM.docx]

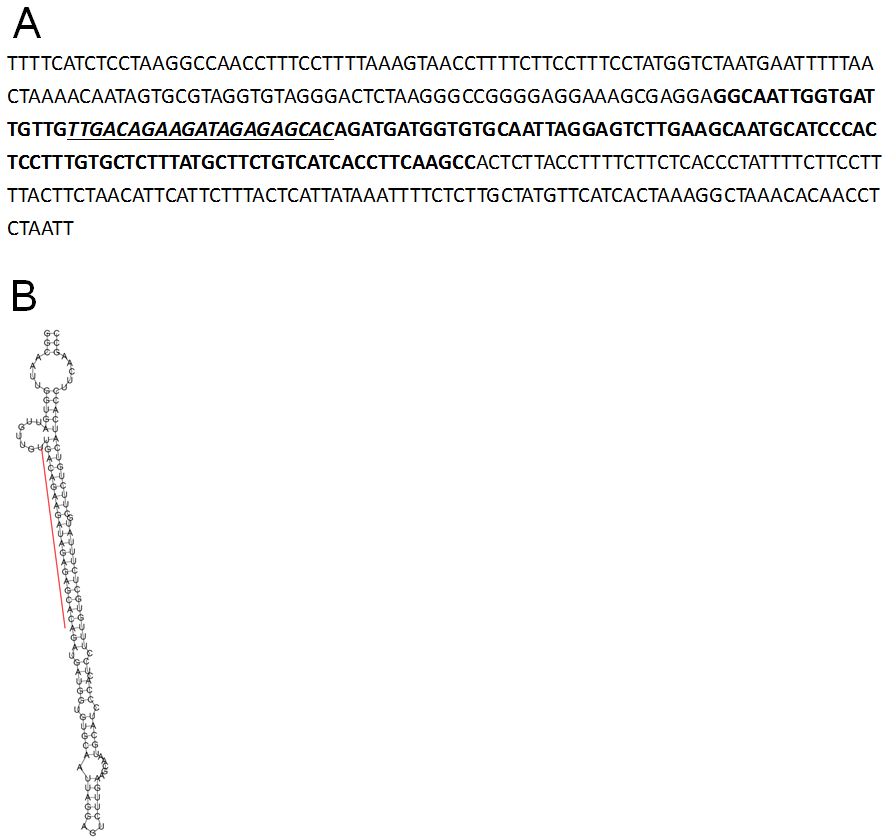


**Additional file 1: GhmiR157 precursor from *Gossypium hirsutum*.** **(A)** Genomic sequence containing GhmiR157 precursor. The underline indicates mature miR157 sequence. **(B)** The secondary structure of GhmiR157 precursor. The red line indicates mature miR157 sequence.
