# Supplementary material for: MicroRNA 157-targeted SPL genes regulate floral organ size and ovule production in cotton
Source: BMC Plant Biol. 2017 Jan 10;17:7. doi: 10.1186/s12870-016-0969-z (PMC5223427; doi:10.1186/s12870-016-0969-z)
Supplement: Additional file 2: — Positive test for 35S::GhmiR157 transformants. (A) Southern blot of 35S::GhmiR157 transformants. (B) PCR analysis of 35S::GhmiR157 transformants. Control, nontransgenic plant segregated from 35S::GhmiR157 transgenic lines in cotton. The numbers 11, 12, 33, 35, 37, 38, 40 represent different 35S::GhmiR157 transgenic lines in cotton. (DOCX 287 kb) [file 12870_2016_969_MOESM2_ESM.docx]

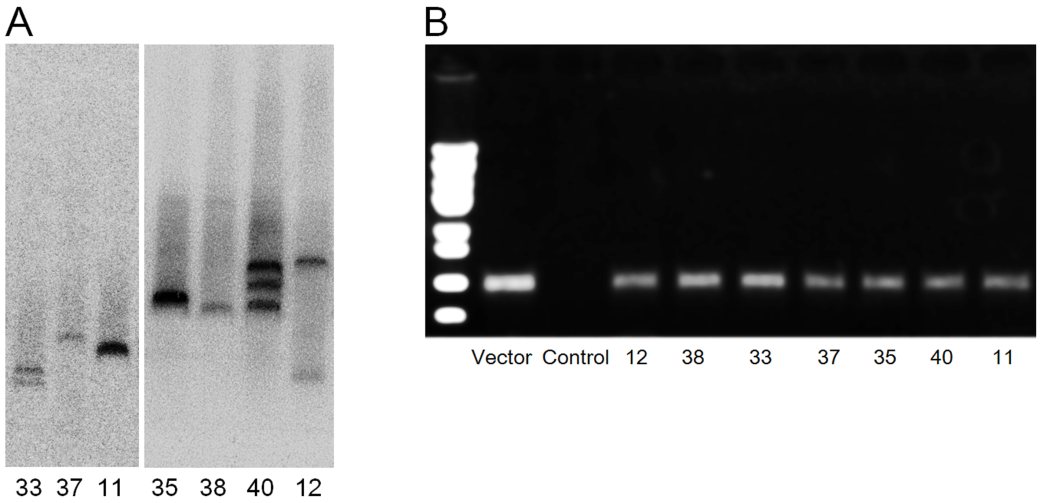


**Additional file 2: Positive test for 35S::*GhmiR157* transformants.** **(A)** Southern blot of 35S::*GhmiR157* transformants. **(B)** PCR analysis of 35S::*GhmiR157* transformants. Control, nontransgenic plant segregated from 35S::*GhmiR157* transgenic lines in cotton. The numbers 11, 12, 33, 35, 37, 38, 40 represent different 35S::*GhmiR157* transgenic lines in cotton.
