# Supplementary material for: MicroRNA 157-targeted SPL genes regulate floral organ size and ovule production in cotton
Source: BMC Plant Biol. 2017 Jan 10;17:7. doi: 10.1186/s12870-016-0969-z (PMC5223427; doi:10.1186/s12870-016-0969-z)
Supplement: Additional file 3: — Over-expressing GhmiR157 precursor leads to more vegetative branches. (A-E) The pictures of plants at squaring stage in trial field from Control (A), OV12 (B), OV38 (C), OV33 (D), OV35 (E). (F and G) Plants at boll opening stage in field from Control (F) and OV12 (G). (H) Measurement of vegetative branches at squaring stage in green house. Control, nontransgenic plant segregated from 35S::GhmiR157 transgenic lines. OV12, 38, 33, 37 and 35, independent 35S::GhmiR157 transgenic lines. Different letters indicate statistically significant differences at P < 0.05 based on analysis of variance (ANOVA) (Tukey’s multiple comparison test). (DOCX 748 kb) [file 12870_2016_969_MOESM3_ESM.docx]

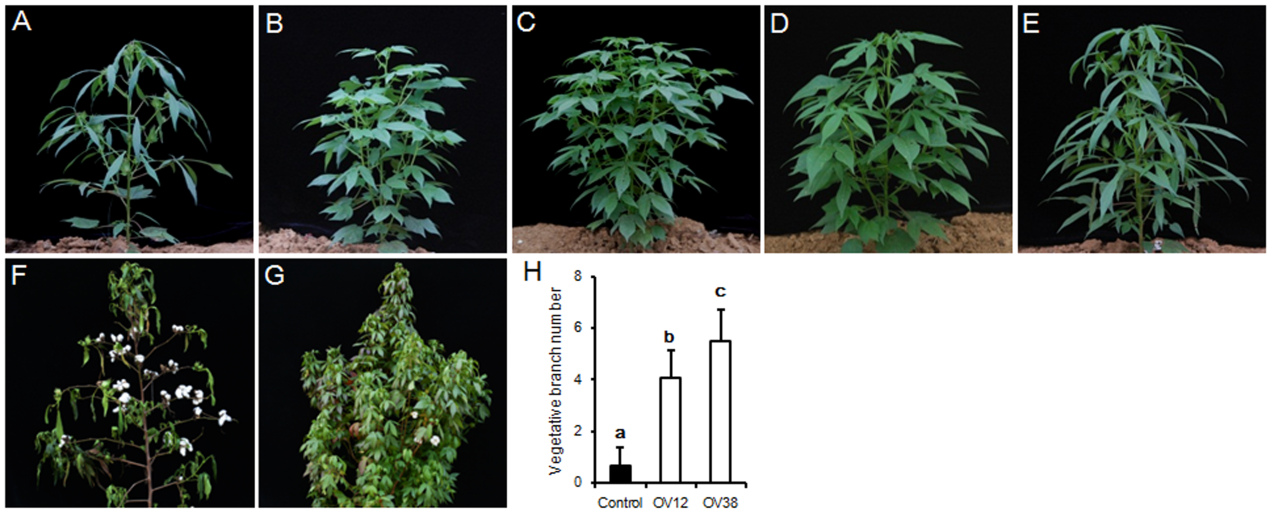


**Additional file 3:** **Over-expressing *GhmiR157* precursor leads to more vegetative branches.** **(A-E)** The pictures of plants at squaring stage in trial field from Control **(A)**, OV12 **(B)**, OV38 **(C)**, OV33 **(D)**, OV35 **(E)**. **(F and G)** Plants at boll opening stage in field from Control **(F)** and OV12 **(G)**. **(H)** Measurement of vegetative branches at squaring stage in green house. Control, nontransgenic plant segregated from 35S::*GhmiR157* transgenic lines. OV12, 38, 33, 37 and 35, independent 35S::*GhmiR157* transgenic lines. Different letters indicate statistically significant differences at *P* < 0.05 based on analysis of variance (ANOVA) (Tukey’s multiple comparison test).
