# Supplementary material for: MicroRNA 157-targeted SPL genes regulate floral organ size and ovule production in cotton
Source: BMC Plant Biol. 2017 Jan 10;17:7. doi: 10.1186/s12870-016-0969-z (PMC5223427; doi:10.1186/s12870-016-0969-z)
Supplement: Additional file 4: — Over-expressing GhmiR157 precursor suppressed reproductive organs development. (A) qRT-PCR of mature miR157 expression in floral buds. R.E.L., the relative expression levels calculated using HISTONE3 (AF024716) as a control. The error bars indicate the standard deviation of three biological replicates. Different letters indicate statistically significant differences at P < 0.05 based on analysis of variance (ANOVA) (Tukey’s multiple comparison test). (B-F) Images of flowers (B), stamens and stigmas (C), ovaries after removing the valves (D), 30 DPA bolls (E) and mature bolls (F). (G) The size of floral organs in WT and over-expressing GhmiR157 lines. Values are shown as the mean ± standard deviation. In each column, values with different letters are significantly different based on Tukey’s multiple comparison test (P < 0.05). OV12, 38, 33 and 35, independent 35S::GhmiR157 transgenic lines. WT, wild type (Gossypium hirsutum cv. YZ1). Control, nontransgenic plant segregated from 35S::GhmiR157 transgenic lines. (DOCX 400 kb) [file 12870_2016_969_MOESM4_ESM.docx]

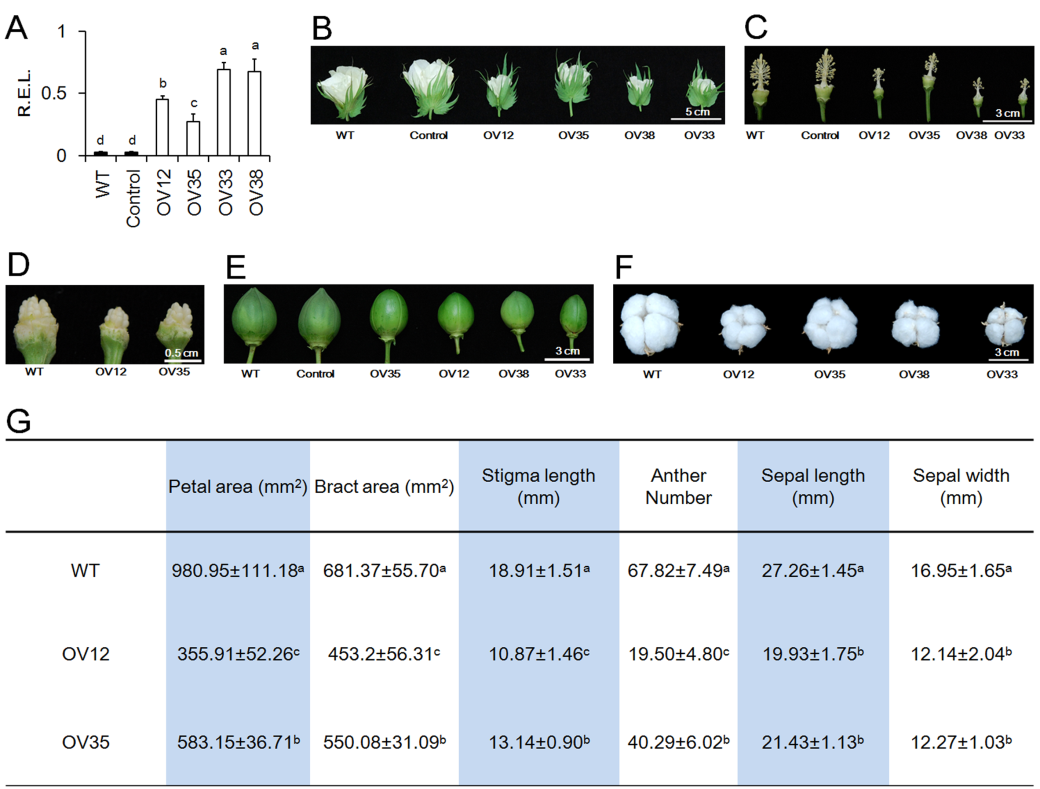


**Additional file 4:** **Over-expressing *GhmiR157* precursor suppressed reproductive organs development.** (A) qRT-PCR of mature miR157 expression in floral buds. R.E.L., the relative expression levels calculated using *HISTONE3* (AF024716) as a control. The error bars indicate the standard deviation of three biological replicates. Different letters indicate statistically significant differences at *P* < 0.05 based on analysis of variance (ANOVA) (Tukey’s multiple comparison test). (B-F) Images of flowers (B), stamens and stigmas (C), ovaries after removing the valves (D), 30 DPA bolls (E) and mature bolls (F). (G) The size of floral organs in wild type and over-expressing *GhmiR157* lines. Values are shown as the mean ± standard deviation. In each column, values with different letters are significantly different based on Tukey's multiple comparison test (*P* < 0.05). OV12, 38, 33 and 35, independent 35S::*GhmiR157* transgenic lines. WT, wild type (*Gossypium hirsutum* cv. YZ1). Control, nontransgenic plant segregated from 35S::*GhmiR157* transgenic lines.
