# Supplementary material for: MicroRNA 157-targeted SPL genes regulate floral organ size and ovule production in cotton
Source: BMC Plant Biol. 2017 Jan 10;17:7. doi: 10.1186/s12870-016-0969-z (PMC5223427; doi:10.1186/s12870-016-0969-z)
Supplement: Additional file 8: — Unrooted phylogram of all SPL genes in Gossypium hirsutum and Arabidopsis. Unrooted phylogram was performed by MEGA6 software based on the neighbor-joining algorithm. The red lines indicate the subfamily members are candidates of miR157 targets predicted through psRNATarget website tool [69]. (DOCX 48.2 kb) [file 12870_2016_969_MOESM8_ESM.docx]

**Additional file 5:** **Unrooted phylogram of all *SPL* genes in *Gossypium hirsutum* and *Arabidopsis*.** Unrooted phylogram was performed by MEGA6 software based on the neighbor-joining algorithm. The red lines indicate the subfamily members are candidates of miR157 targets predicted through psRNATarget website tool (http://plantgrn.noble.org/psRNATarget/).
