# Supplementary material for: MicroRNA 157-targeted SPL genes regulate floral organ size and ovule production in cotton
Source: BMC Plant Biol. 2017 Jan 10;17:7. doi: 10.1186/s12870-016-0969-z (PMC5223427; doi:10.1186/s12870-016-0969-z)
Supplement: Additional file 9: — Quantification analysis of MADS-box genes in ectopic-expressing GhmiR157 precursor lines and wild type. (A-D) qRT-PCR of MADS-box genes in inflorescence. E4 and E6 indicate 35S::GhmiR157 transgenic lines in Arabidopsis. R.E.L., the relative expression levels calculated using AtACT7 (AT5G09810.1) as a control. The error bars indicate the standard deviation of four biological replicates. (DOCX 88.3 kb) [file 12870_2016_969_MOESM9_ESM.docx]

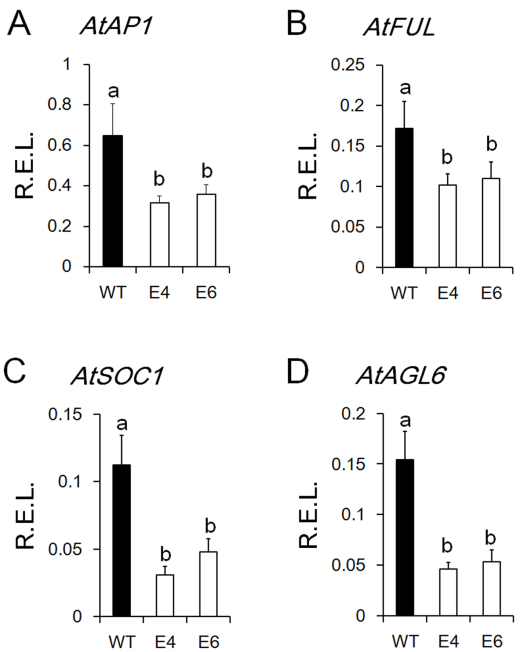


**Additional file 6:** **Quantification analysis of MADS-box genes in ectopic-expressing GhmiR157 precursor lines and wild type.** (A-D) qRT-PCR of MADS-box genes in inflorescence. E4 and E6 indicate 35S::*GhmiR157* transgenic lines in *Arabidopsis*. R.E.L., the relative expression levels calculated using *AtACT7* (AT5G09810.1) as a control. The error bars indicate the standard deviation of four biological replicates. Different letters indicate statistically significant differences at *P* < 0.05 based on analysis of variance (ANOVA) (Tukey’s multiple comparison test).
